# Gadolinium Oxide Nanoparticles Induce Toxicity in Human Endothelial HUVECs via Lipid Peroxidation, Mitochondrial Dysfunction and Autophagy Modulation

**DOI:** 10.3390/nano10091675

**Published:** 2020-08-26

**Authors:** Mohd Javed Akhtar, Maqusood Ahamed, Hisham Alhadlaq

**Affiliations:** 1 King Abdullah Institute for Nanotechnology, King Saud University, Riyadh 11451, Saudi Arabia; mahamed@ksu.edu.sa; 2 Department of Physics and Astronomy, College of Sciences, King Saud University, Riyadh 11451, Saudi Arabia; hhadlaq@ksu.edu.sa

**Keywords:** oxidative stress, ROS, autophagy, lysosome, necrosis

## Abstract

In spite of the potential preclinical advantage of Gd_2_O_3_ nanoparticles (designated here as GO NPs) over gadolinium-based compounds in MRI, recent concerns of gadolinium deposits in various tissues undergoing MRI demands a mechanistic investigation. Hence, we chose human to measure umbilical vein endothelial cells (HUVECs) that line the vasculature and relevant biomarkers due to GO NPs exposure in parallel with the NPs of ZnO as a positive control of toxicity. GO NPs, as measured by TEM, had an average length of 54.8 ± 29 nm and a diameter of 13.7 ± 6 nm suggesting a fiber-like appearance. With not as pronounced toxicity associated with a 24-h exposure, GO NPs induced a concentration-dependent cytotoxicity (IC_50_ = 304 ± 17 µg/mL) in HUVECs when exposed for 48 h. GO NPs emerged as significant inducer of lipid peroxidation (LPO), reactive oxygen species (ROS), mitochondrial membrane potential (MMP) and autophagic vesicles in comparison to that caused by ZnO NPs at its IC_50_ for the same exposure time (48 h). While ZnO NPs clearly appeared to induce apoptosis, GO NPs revealed both apoptotic as well as necrotic potentials in HUVECs. Intriguingly, the exogenous antioxidant NAC (N-acetylcysteine) co-treatment significantly attenuated the oxidative imbalance due to NPs preventing cytotoxicity significantly.

## 1. Introduction

Advances in nanotechnology have yielded nanoparticles (NPs; defined as particles having size less than 100 nm, at least, in one dimension) that are under exploration in various applications ranging from catalysis, biosensing, imaging, cancer therapy, DNA, drug delivery and enzyme immobilization [1,2]. NPs with potential biomedical applications are most likely to interact with human posing potential health hazard. One such applications of NPs is in the diagnosis of diseases using magnetic resonance imaging (MRI). At the core of MRI are the different kinds of contrast agents. Most contrast agents in MRI are based on gadolinium element (or gadolinium compounds) [3,4,5]. Moreover, in pursuit of improving detection capability in MRI, NPs based on gadolinium element are under research in preclinical models [6,7,8]. Whatever the form of gadolinium used in MRI, concerns are increasing due to recent reports about accumulation of gadolinium in many tissues of patients undergoing MRI [9,10]. The gadolinium element has been reported to enter the circulation and to deposit in the liver, breast, kidney, lung and neural tissues [4]. Previously believed to be risk-free, gadolinium has recently been shown to damage vasculature, allowing gadolinium deposition in various tissues [11]. Analysis of post-mortem brain tissue has confirmed gadolinium deposition under the endothelial walls and the neuronal interstitium [12,13]. Moreover, there are direct evidences of gadolinium travel to, and deposit in the brain, therein leading to impairment in locomotor function of the central nervous system in mice fed gadolinium-containing food pellets [14]. Gadolinium deposition has also reported in the periodontal ligament of mice, resulting in reduced renal function [15].

Endothelial cells, therefore, are recommended in advancing the mechanism of toxicity that could be elicited due to the entry of NPs in the circulation and circulatory vasculature [16]. Investigations of the interaction and response of endothelial cells with NPs used especially in biomedical science are necessary to understand the dynamics and inflammation associated with potential damages [17]. Endothelial cells that line the vasculature allowing transport of nutrients and waste to and from organs justifies their use in the nanotoxicology [18]. Moreover, endothelial cells constitute an important site of endocytosis and autophagy for infiltrating bacteria, viruses and other foreign particles [19]. Human umbilical vein endothelial cells (HUVECs) are proven model for extending mechanistic research in vitro, corroborating endothelial cell dysfunction and potential damage occurring to the integrity of blood vessels [20,21]. To the best of our knowledge, studies on bio-response of GO NPs in endothelial cells including HUVECs are lacking. Therefore, this study was designed to understand the effect and mechanism of toxicity due to GO NPs that could shed light behind vasculature damages as reported above. In this study, NPs of rare-earth GO were synthesized via a chemical route. Transmission electron microscope (TEM) determined the size and shape of GO NPs. Energy dispersive spectrum (EDS) analysis and X-ray diffraction (XRD) confirmed the NP’s chemical composition and crystalline texture of GO NPs, respectively. In order to understand the mechanism behind the potential toxicity due to GO NPs, membrane integrity and intracellular oxidative imbalances were assessed. Mitochondrial functions are strongly affected by oxidative stress that set the pathway of autophagy, apoptosis and necrosis [22]. Mitochondrial function, therefore, was determined by JC-1 under a fluorescence microscope. As discussed, NPs of rare-earth metal oxides are emerging agents for disrupting autophagic processes. The mechanism of autophagy was investigated by the combined use of LysoTracker (LTR) that traces lysosomes, and other intracellular acidic vesicles and monodansylcadaverine (MDC) that track critical events during autophagy. Triple-staining comprising of PI, Hoechst and annexinV—as well as caspase-3 activity—was used to decipher the mode of cell death involved due to GO NPs. As they have been established as toxic NPs in many epithelial cells [23] and endothelial cells [24], ZnO NPs were used appropriately as a positive control of toxicity. GO NPs exhibited significantly high tendency of inducing membrane damage, oxidative stress, mitochondrial dysfunction and autophagy than ZnO NPs in HUVEC cells at their respective IC_50s_. While ZnO NPs clearly induced a GSH-depletion-dependent apoptosis in HUVECs, NPs of GO appeared to induce both apoptosis and necrosis—similar to silica NPs with heterogeneity in size and structure [21,25].

## 2. Materials and Methods

### 2.1. Chemicals and Reagents

Fetal bovine serum, penicillin–streptomycin, calcein-AM, BODIPY and LTR (LysoTracker™ Red DND-99) were purchased from Invitrogen Co. (Carlsbad, CA, USA). Gd(NO_3_)_3_∙6H_2_O and glycine, DMEM F-12, MTT [3-(4,5-dimethyl thiazol-2-yl)-2,5-diphenyl tetrazolium bromide], NADH, pyruvic acid, perchloric acid, DCFH-DA, MDC (monodansylcadaverine), autophagy kit, JC-1 (5,5′,6,6′-tetrachloro-1,1′,3,3′-tetrethyl-imidacarbocyanine iodide), Hoechst (bisbenzimide H 33,342 trihydrochloride), PI (3,8-diamino-5-[3-(diethylmethylammonio)propyl]-6-phenylphenanthridinium diiodide), GSH, o-phthalaldehyde (OPT), hank’s balanced salt solution (HBSS), N-acetylcysteine, L-buthionine-sulfoximine and Bradford reagent were obtained from Sigma-Aldrich (Sigma-Aldrich, MO, USA). AnnexinV-FITC apoptosis/necrosis kit was purchased from BD Biosciences (Franklin Lakes, NJ, USA). Ultrapure water was prepared from a Milli-Q system (Millipore, Bedford, MA, USA). All other chemicals used were of reagent grade.

### 2.2. Preparation and Characterization of Gadolinium Oxide Nanoparticles (GO NPs)

Nanoparticles of GO NPs were synthesized by thermal decomposition method as described by Kuzníková et al. [26]. Accordingly, an aqueous solution of gadolinium nitrate (Gd(NO_3_)_3_∙6H_2_O) and glycine (NH_2_CH_2_COOH) each with a concentration of 0.5 M were mixed with continuous stirring for 4 h. The complex formed in this process was dried at 120 °C for 1 h and calcined at 600 °C for 1 h. Thermal decomposition of the complex that occurred at about of (250 ± 10 °C) was followed by milling in air atmosphere in alumina crucibles for 1 h. Other components such as N_2_, CO_2_ and H_2_O in the complex were evaporated in gaseous state. The resultant GO NPs were characterized by various parameters. The shape and size of the nanocrystals were evaluated by field emission transmission electron microscopy (FETEM, JEM-2100F, JEOL, Inc., Tokyo, Japan). The surface morphology of nanocrystals was captured by scanning electron microscope (FESEM, JSM-7600F, JEOL, Inc., Tokyo, Japan). Energy dispersive spectrum (EDS) analysis was used to confirm the chemical composition of nanocrystals. X-ray diffraction (XRD) was carried and confirmed by the method described by Kuzníková et al. [26].

### 2.3. Cell Culture and Treatment with GO NPs

Human umbilical vein endothelial cells (HUVECs) (ATCC, Manassas, VA, USA) were maintained in DMEM-F12 supplemented with 10% fetal bovine serum, endothelial growth supplement (CADMEC, Cell Applications, Inc., San Diego, CA, USA) and antibiotics 100 U/mL penicillin and 100-µg/mL streptomycin at 37 °C in a humidified 5% CO_2_ incubator (HERA Cell 150i, Thermo Fisher Scientific, Waltham, MA, USA). The cells were passaged every 3–4 days. GO NPs were suspended directly in culture media, ultrasonicated for 5 min (ultrasonic cleaner 8891, Cole-Parmer, 625 Bunker Court Vernon Hills, IL, USA) and diluted to appropriate concentration in 10 mL of media in sterile tubes. Cells seeded before 24 h in culture vessels were immediately exposed. Untreated cell group served as control in each experiment. The 48-h exposure time was chosen for investigation since responses at 24-h exposure were not as pronounced as were at 48 h. Therefore, all the data presented here is that of 48-h exposure. Biochemical outcomes due to toxic ZnO NPs were compared with that of GO NPs in order to understand the underlying biochemical mechanism of toxicity in greater depth.

### 2.4. Cell Viability Assays

An MTT assay was carried out to measure cell viability according to the protocol described by Mosmann [27] with minor modifications. Approximately 1 × 10^4^ HUVECs were seeded in a 96-well plate with a clean and flat bottom. On the following day, cells were treated with NPs. After the exposure period (48 h), the MTT assay was performed by measuring absorbance in a plate reader (Synergy HT, Bio-Tek, Winooski, VT, USA) at 570 nm from a clear NPs-free supernatant obtained after centrifuging the treated plate. Cell viability is given in the percentage of control cells, assuming 100% cell viability in the control.

### 2.5. Evaluation of Cell Membrane Integrity

Cell membrane integrity was determined by conventional methods as given below followed by direct observation of cells under microscope. Lactate dehydrogenase (LDH) release was measured by taking 100 µL centrifuged culture media and mixing it in a total volume of 3.0 mL of LDH assay cocktail (100 µL of 6-mM Na-pyruvate, 100 µL of 0.4-mM NADH and 2.7 mL 0.1-M K-phosphate buffer, pH 7.4) [28]. A decrease in the absorbance of NADH at 340 nm recorded for 3 min at 25 °C using a spectrophotometer (Genesys 10 Bio, Thermo Fisher Scientific, Madison, WI, USA) was converted into LDH activity (IU/L) released in the culture media. The basic mechanism of membrane damage caused by lipid peroxidation (LPO) in fatty acids was quantified by the method of Ohkawa et al. [29]. Results were calculated as nmol TBARS/mg of cellular protein using 1.56 × 10^5^ M^−1^ cm^−1^ as molar extinction coefficient of MDA–TBA adduct. LPO determined by TBARS method was confirmed by direct observation of cells labeled with lipophilic C11-BODIPY581/591 probe (Life Technologies-Invitrogen, Carlsbad, CA, USA) as described elsewhere [30,31]. This is a ratio metric dye which is readily incorporated in membrane bilayers of cells that emits uniform red fluorescence in non-oxidized state and green fluorescence in oxidized state in proportional to the amount of LPO [32]. Briefly, cells in 12-well culture plate were labeled with freshly prepared C11-BODIPY581/591 probe in HBSS at the final concentration of 2 µM and incubated for 60 min in the dark. Cells were carefully washed three times with HBSS to remove excess dye that interfere particularly during green emission and imaged under a fluorescence microscope (Leica DMi8 manual, Wetzlar, Germany) using appropriate filters.

### 2.6. Detection of Intracellular ROS

The generation of intracellular ROS was measured using 2′,7′-dichlorofluorescin diacetate (DCFH-DA) probe [33]. Cells were seeded in 96-well plate and treated with NPs as for MTT, but in black plates with transparent bottom. When the treatment period was over the medium was aspirated off and 100 µL of DCFH-DA working solution in HBSS at the final concentration of 50 µM was incubated for 45 min. Then each well was washed twice with cold PBS to remove excess dye from the aqueous media and DCF fluorescent intensity was measured at the emission 528 nm in a plate reader (Synergy HT, Bio-Tek, Winooski, VT, USA).

### 2.7. Quantification of Intracellular GSH

The cellular content of GSH was quantified according to the method given by Hissin and Hilf [34]. Cells were washed, collected by scrapping and washed with PBS two times. Then, cells were lysed in aqueous solution of 0.1% deoxycholic acid plus 0.1% sucrose for 2 h that included 3 cycle of freeze–thaw and centrifuged at 10,000× *g* for 10 min at 4 °C. The supernatant was precipitated in 1% perchloric acid and centrifuged at 10,000× *g* for 5 min at 4 °C. A 20 µL from the protein precipitated sample was mixed with 160 µL of 0.1-M K-phosphate, 5-mM EDTA buffer, pH 8.3 and 20 µL o-phthalaldehyde (OPT, 1-mg/mL in methanol) in black 96-well plate. After 2.5 h of incubation at room temperature in the dark, fluorescence was measured at an emission wavelength of 460 nm (Synergy HT, Bio-Tek, Winooski, VT, USA) along with similarly prepared standards of GSH. Protein was estimated from un-precipitated supernatant and data converted to GSH nmol/mg protein.

### 2.8. Determination of Mitochondrial Membrane Potential

Mitochondrial membrane potential (MMP) in control and treated cells was determined by JC-1. In functional mitochondria, JC-1 spontaneously forms complexes known as J-aggregates that produce intense and discrete red fluorescence. In dysfunctional mitochondria, JC-1 remains in the monomeric form that emits bright green fluorescence [35]. When treatment period was over, media was aspirated off from each well and labeled with 5-µM JC-1 in HEPES-buffered HBSS for 20 min. Images of JC-1 monomer and aggregate in control and treated cells were captured by a fluorescence microscope (Leica DMi8 manual, Wetzlar, Germany). Colocalization of the two states of JC-1 was demonstrated by merging of the two images in ImageJ software (NIH, Bethesda, MD, USA).

### 2.9. Measurement of Autophagic/Acidic Vesicles

Autophagy was measured by a commercial kit (MAK-138, Sigma-Aldrich, St. Louis, MO, USA) as well by fluorescence microscopy with LysoTracker (LTR) and monodansylcadaverine (MDC) probes. LTR dyes are specific for the detection of acidic vesicular organelles represented predominantly by lysosomes and autolysosomes that are fusion product of autophagosomes with lysosomes [36]. MDC is another marker of acidic vesicles which is used to monitor late-stage autophagy event that occurs after fusion of lysosomes with autophagosome forming autolysosome [37,38]. In this study, autophagy was tracked by multiplexing cells with MDC and LTR in tandem because of their distant emission fluorescence spectra. Final labeling conditions of MDC and LTR were 50 mM and 1 µM, respectively for 40 min in HUVECs in 12-well plate. Imaging was conducted using excitation filter for violet (detecting blue colored MDC emission) and green filter (detecting red colored LTR emission) under a fluorescence microscope (Leica DMi8 manual, Wetzlar, Germany) after carefully washing plates three times with cold HBSS buffer.

### 2.10. Apoptosis/Necrosis Detection via Triple Staining and Caspase-3 Activity

A triple staining approach was adopted according to the convention described [39] in which each defined combination of fluorescence demonstrates different status of cell health. A universal cell permeable dye Hoechst 33442 was used to trace healthy cells (marked by only blue fluorescence), apoptotic/necrotic cells marked by colocalized combination of green colored annexinV staining and red colored PI staining [40,41]. Necrosis from apoptosis was differentiated by preferential uptake of PI over annexinV, pattern of chromatin condensation and nuclear perimeter dilation [42,43]. First cells were labeled with annexinV for 20 min and then washed two times with annexin binding buffer removing excess annexinV-FITC dye. Then, Hoechst 33342 and PI were used at the final concentration of 1 µg/mL for each reagent and left incubated during imaging. This is an inexpensive and more dependable method which was successfully applied in our previous publication [31].

Activity of caspase-3 enzyme was determined from cell lysate of control and treated cells. In brief, 5 × 10^4^ HUVECs were seeded in T25 culture flasks and treated with NPs of GO and ZnO. A reaction mixture containing 30 µL of cell lysate, 20 µL of Ac-DEVDAFC (caspase-3 substrate), and 150 µL of protease reaction buffer (50-mM HEPES, 1-mM EDTA and 1-mM DTT, pH 7.2) was incubated. The fluorescence of the reaction mixture was measured at every 5 min intervals for 15 min at excitation/emission wavelengths of 430/535-nm using a microplate reader (Synergy HT, Bio-Tek, Winooski, VT, USA). The 7-amido-4- trifluoromethylcoumarin (AFC) standard ranging from 5 µM to 15 µM was prepared and its fluorescence was recorded for calculation of caspase-3 activity in pmol AFC released/min/mg protein.

### 2.11. Protein Estimation

The total protein content was measured by a convenient BCA protein assay kit from Sigma-Aldrich as per instructions.

### 2.12. Statistics

ANOVA (one-way analysis of variance) followed by Dunnett’s multiple comparison tests was employed for statistical analysis of results. For a particular set of the experiment, a burst of images was captured at the constant exposure of time, gain, saturation and gamma. Calculation of corrected total cellular fluorescence (CTCF) was conducted in ImageJ software (NIH, Bethesda, MD, USA). Using the region of interest (ROI) manager command, a reasonably constant area was restored via ‘restore selection’ command to all images once opened in an ImageJ session. CTCF was calculated by subtracting the mean of background (without cell) fluorescence from the mean of cellular fluorescence (i.e., mean integrated density). The scale bar in images was set using ImageJ after adjusting the scale in terms of pixels/micron and then saving all images in a JPEG format. Representative images (captured by Leica DFC450 camera, Wetzlar Germany) from three independent experiments (*n* = 3) are shown for the particular experimental group. Data represented are means ± SD of three identical experiments (*n* = 3) made in triplicates in all of biochemical and imaging experiments. Statistical significance was attributed at *p* < 0.05.

## 3. Results

### 3.1. Physicochemical Characterization of GO NPs

**Figure 1:**
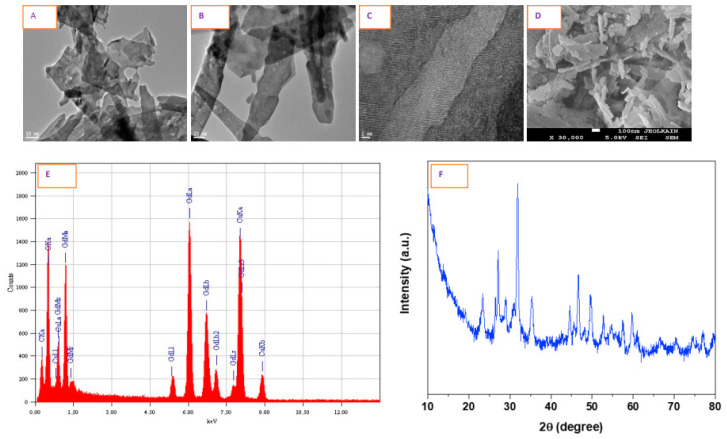
Shape and size of gadolinium oxide (GO) nanoparticles (NPs) characterized by TEM image captured at (**A**) 50 nm and (**B**) 20 nm. High resolution TEM image captured at (**C**) 2 nm depicts crystal plane. (**D**–**F**) SEM, EDS and XRD images of GO NPs, respectively.

The size of GO NPs was calculated from over 100 particles in random fields of TEM view. TEM images measured a length of 60–110 nm and a diameter of 9–23 nm, suggesting an average aspect ratio of over 4. GO NPs, thus, appeared fibrous in shape (Figure 1A,B). Texture shown in high resolution TEM (HRTEM) image (Figure 1C) confirmed the planes atypical of crystal structures. SEM image (Figure 1D) again confirmed the nanofiber-like morphology of GO NPs with high heterogeneity. Elemental dispersive spectrum (Figure 1E) analysis confirmed gadolinium and oxygen element in GO NPs. XRD is given in Figure 1F which matches with XRD pattern of GO NPs reported by Kuzníková et al. [26]. The dispersibility of GO NPs was higher in the complete culture media than it was in water as denoted by hydrodynamic sizes and zeta potentials. The physicochemical properties are shown in Table 1.

### 3.2. GO NPs-Induced Concentration-Dependent Cytotoxicity in HUVECs

**Figure 2:**
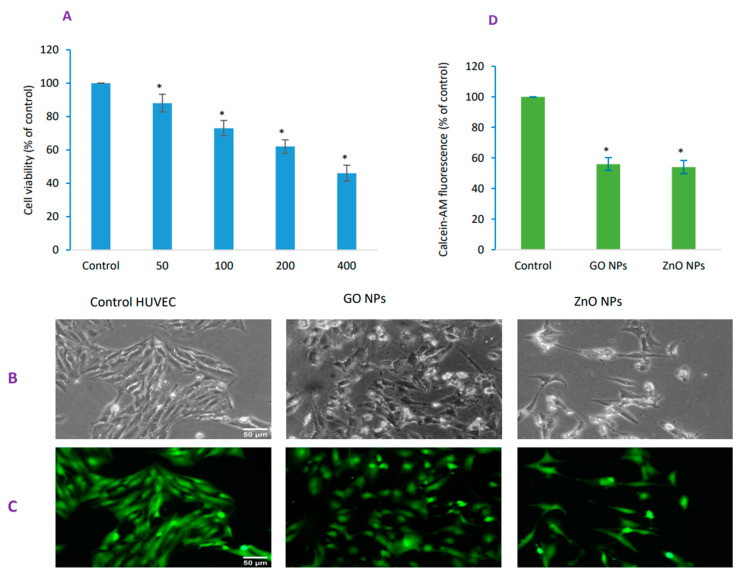
Potential cytotoxicity in human umbilical vein endothelial cells (HUVECs) due to GO NPs was demonstrated by (**A**) MTT biochemical assays followed by imaging same cells under settings of (**B**) phase-contrast and (**C**) calcein-AM live imaging. (**D**) Corrected total cellular fluorescence (CTCF) plotting of calcein-AM. For concentration-dependent cytotoxicity (IC_50_) calculation, a scatter plot in Microsoft Excel was inserted followed by setting the *Y*-axis to logarithmic. Then a trend line was selected and ‘exponential’ picked. Then ‘display equation’ was used in calculating ICs. IC_50s_ calculations were further verified and confirmed from the online IC_50_ calculator (https://www.aatbio.com/tools/ic50-calculator) provided by AAT BioQuest, Inc. (CA 94085, USA). Scale bar represents 50 µm (20× objective). * Statistically significant difference than the controls (*p* < 0.05).

Cell viability, as evaluated by MTT assay, was decreased to 88, 73, 62 and 46%, respectively due to concentrations of 50, 100, 200 and 400 µg/mL of GO NPs suggesting a concentration-dependent toxicity when exposed for 48 h (Figure 2A). Based on MTT data, IC_50_ of GO NPs for a 48-h exposure in HUVECs was calculated to be 304 ± 17 µg/mL according to method explained in Figure 2 legend. IC_50_ for ZnO NPs came out to be 43 ± 4 µg/mL in a similar condition. Cell morphology due to exposure of IC_50_ of GO NPs and ZnO NPs was determined by microscopy under phase-contrast (Figure 2B) and calcein-AM fluorescence (Figure 2C). Recall that healthy cells fluoresce brightly whereas compromised or dead cells fluoresce dim as evidenced in calcein-AM fluorescence image (Figure 2C) and calcein-AM fluorescence quantification (Figure 2D). Compromised or dead cells due to GO NPs exhibited higher tendency of adherence as compared with ZnO NPs treatment in which dead or damaged cells were easily washed off in subsequent washing protocol. This study was advanced by appropriately chosen ZnO NPs as a positive control because of its high toxicity established [23,24].

### 3.3. GO NPs Caused Significant Damage to the Membrane Integrity

**Figure 3:**
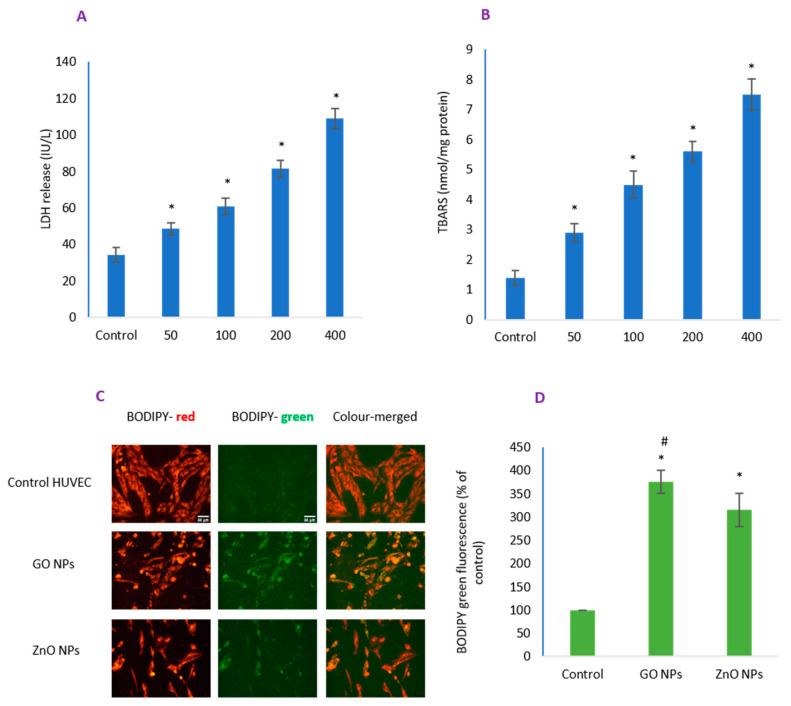
Potential loss of membrane integrity in HUVEC cells due to GO NPs were revealed by (**A**) quantifying LDH release in culture media, (**B**) peroxidation in lipid bilayers poly unsaturated fatty acids, followed by (**C**) imaging of membrane residing BODIPY probe; (**D**) BODIPY CTCF of only green fluorescence is given, as it is the indicator of lipid peroxidation (LPO) while red fluorescence is uniform in control and treated cells. Scale bar represents 50 µm in each image (20× objective). * Statistically significant difference than the controls (*p* < 0.05). #—Significantly high BODIPY fluorescence due to GO NPs compared to ZnO NPs (*p* < 0.05).

LDH and LPO measurements demonstrated a concentration-dependent loss in membrane integrity (Figure 3A,B). The magnitude of damage occurring due to IC_50_ of GO NPs was well correlated in BODIPY imaging (Figure 3C) and its quantitative data (Figure 3D). As in cytotoxicity studies above, cells appeared more adherent in GO NPs treatment than in the treatment of ZnO NPs (see Figure 3C). Collectively, damage to membrane integrity was significantly higher due to GO NPs when compared to that induced by ZnO NPs.

### 3.4. GO NPs Elicited Significant Oxidative Stress

**Figure 4:**
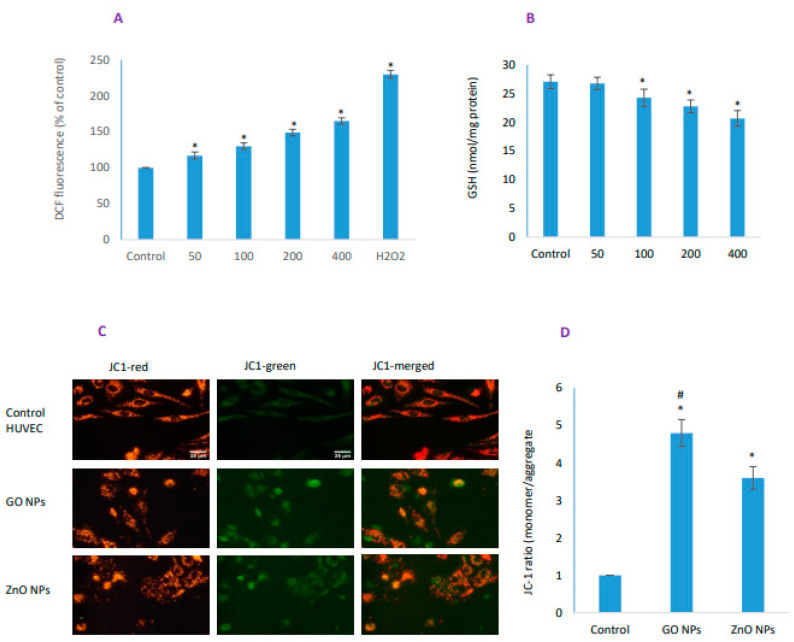
Level of oxidative stress was determined by measuring (**A**) reactive oxygen species (ROS) induction and (**B**) GSH depletion in HUVEC cells. H_2_O_2_ was used as a positive control of oxidant in DCFH-DA probing. MMP was detected by JC-1 in control and treated groups of HUVECs; (**C**) images captured in tandem for JC-1 monomer (green) and JC-1 aggregate (red); (**D**) Quantification of MMP is given as ratio of monomer/aggregate. Scale bar represents 25 µm in each image (40× objective). * Statistically significant difference than the controls (*p* < 0.05). #—Significantly high MMP induction due to GO NPs compared to ZnO NPs (*p* < 0.05).

GO NPs significantly increased intracellular ROS that occurred in a concentration-dependent manner (Figure 4A). It also significantly induced exhaustion of cellular GSH level except 50 µg/mL though the GSH depletion was steeper for ZnO NPs (Figure 4B, also see later Figure 7B). The magnitude of cellular ROS induction was 1.7-fold due to IC_50_ of GO NPs whereas GSH depletion was 1.2-fold when compared with control HUVECs. On the other hand, IC_50_ of ZnO NPs caused 1.5-fold ROS induction and 2.7-fold GSH depletion when compared with the control HUVECs. Mitochondrial damage was assessed by colocalization of red JC-1 aggregate and green JC-1 monomer. It is clear from the qualitative (Figure 4C) and quantitative (Figure 4D) data that both NPs caused significant loss in mitochondrial membrane potential, but MMP was higher for GO NPs than it was for ZnO NPs.

### 3.5. GO NPs Revealed Significantly Higher Autophagy Potential than ZnO NPs

**Figure 5:**
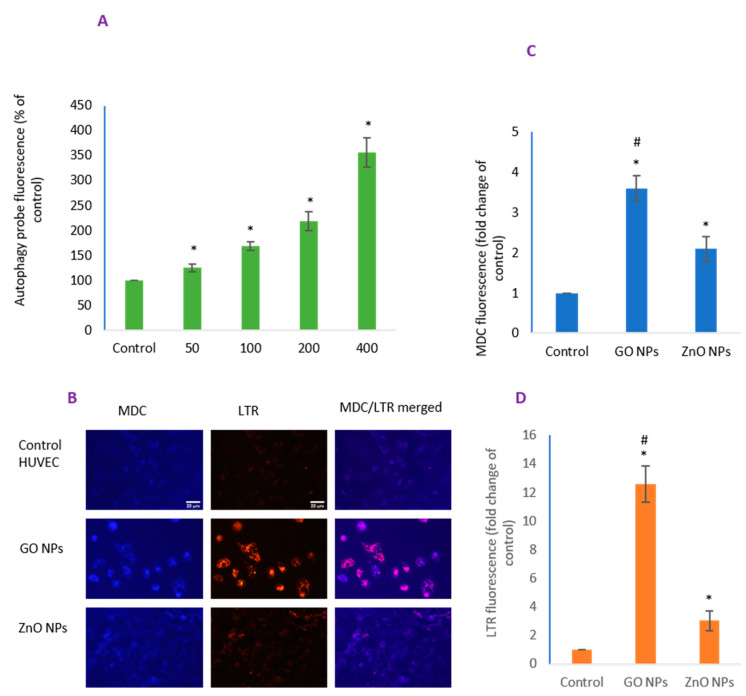
Autophagy was determined by a (**A**) plate reader as well as by (**B**) direct observation under microscopy. Merging of images was carried out in ImageJ software; (**C**,**D**) CTCF for monodansylcadaverine (MDC) and LysoTracker (LTR), respectively. Scale bar represents 25 µm (40× objective). * Statistically significant difference than the controls (*p* < 0.05). #—Significantly high MDC and LTR activity due to GO NPs compared to ZnO NPs (*p* < 0.05).

Autophagy occurred in a concentration-dependent manner for GO NPs when measured by a commercial kit (Figure 5A). Cells colocalized with concurrent labeling with MDC and LTR probe (Figure 5B) demonstrated a higher autophagy potential due to GO NPs than ZnO NPs. Merged images suggest MDC staining, a marker of late stage event in autophagy [44], is highly aligned with the induced LTR fluorescence, a marker of acidic lysosomes, autophagosomes and autolysosomes [45,46].

### 3.6. Cell Death Due to GO NPs Appeared Independent of Apoptosis

**Figure 6:**
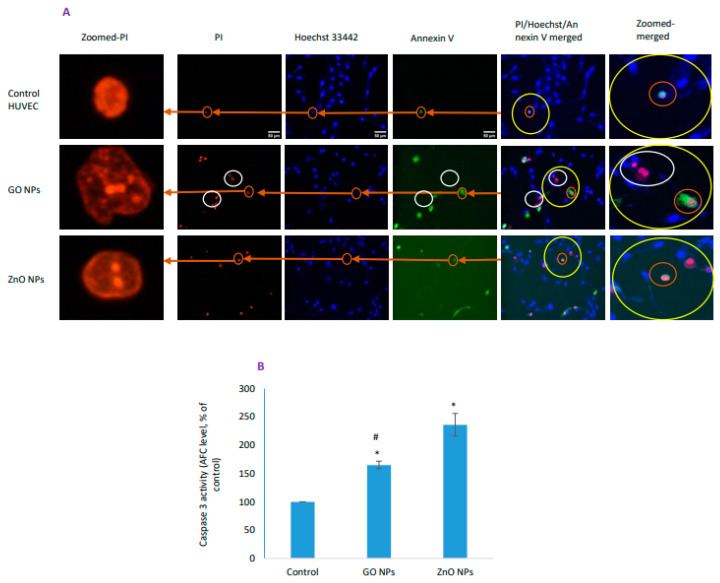
HUVEC cells treated with toxic concentrations of NPs of GO and ZnO for 48 h and apoptosis/necrosis was determined by (**A**) triple-staining and (**B**) caspase-3 activity. Cells were stained with Hoechst 33442 (blue color) that stains nucleus of live or dead cell, PI (red color) that stains nucleus of only dead or dying cell and annexinV (green color) that preferentially stain apoptotic cells. Zoomed images (left) represent equal areas carved out from each of PI images to observe morphology of nucleus in greater detail whereas zoomed out images (right) are carved out from each merged image for a better visualization representing the area equal to yellow circles. Scale bar represents 50 µm (20× objective). * Statistically significant difference than the controls (*p* < 0.05). #—Significantly low caspase-3 activity due to GO NPs compared to ZnO NPs (*p* < 0.05).

Triple staining as explained in Figure 6A suggested GO NPs to induce apoptosis as well as necrosis in contrast to NPs of ZnO that was found to be strong inducer of apoptosis together with caspase-3 data (Figure 6B). In triple staining imaging experiments, two group of cells were marked in GO NPs-treated group. First, cells (encircled white in Figure 6A) were observed that stained with PI but lacked annexinV whereas there were cells that were stained with both PI and annexin. Recall in necroptosis, there are cells that do stain with PI, but still lacks annexinV binding due to lack of initial “find me” and “eat me” signals that are a feature of apoptosis [40] and that PI preferentially enters necrotic cells while is excluded from early apoptotic cells that bind annexinV only [41]. In ZnO NP-treated group, almost all cells were found to stain with PI and annexinV simultaneously. Another method of differentiating apoptosis from that of necrosis is based on nuclear detailing. Cells undergoing necroptosis are marked by exhibiting significantly increased nuclear shape perimeter while secondary staining with annexinV is due to passive entry [47] as is clear in zoomed images (see Figure 6A). Zoomed images represent equal areas carved from each of PI images to observe morphology of a nucleus of damaged cells in greater detail. In zoomed images, damaged nuclei from each treatment group are increased in size with different degree of fragmentation and chromatin condensation (signs of apoptosis in case of ZnO NP while apoptosis-independent in case of GO NP). In contrast to treatment groups, nucleus from control group is more compact with much lesser in nuclear perimeter and that lack the pattern of chromatin fragmentation with either treatment group. 

### 3.7. Inhibitory Effect of NAC on Induced Oxidative Stress and Cytotoxicity Due to NPs

**Figure 7:**
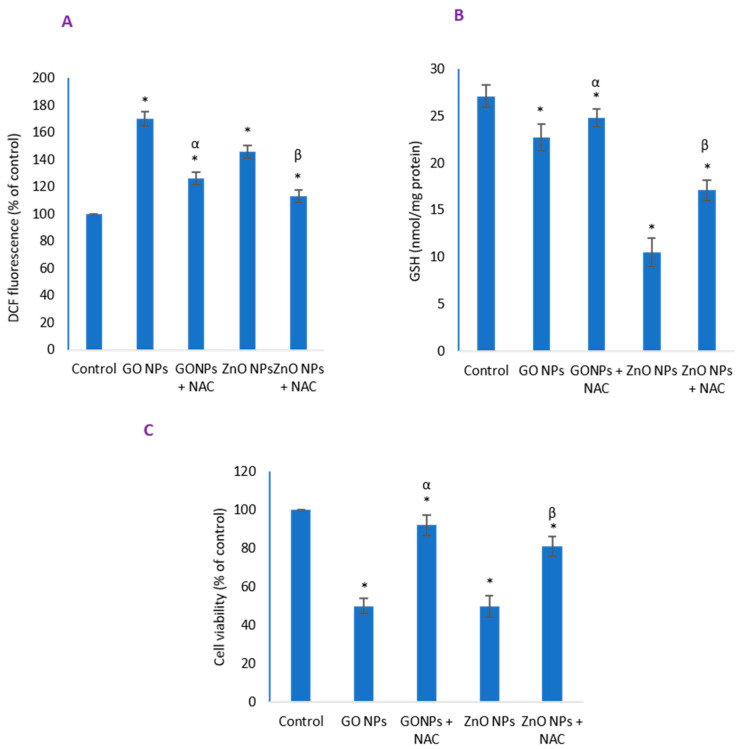
Inhibitory effect of N-acetylcysteine (NAC) cotreatment on (**A**) ROS-induction, (**B**) GSH-depletion—and consequently, (**C**) cytotoxicity that would otherwise be induced by NPs alone. * Statistically significant difference than the controls (*p* < 0.05). α and β denote significant preventive potential of NAC on ROS generation, GSH decline and cytotoxicity due to GO NPs and ZnO NPs, respectively (*p* < 0.05).

Antioxidant N-acetylcysteine (NAC, 2 mM) was applied to cells 30 min before NPs treatment to test the potential reduction, if any, of induced oxidative stress. ROS that was induced to 1.7-fold was reduced to 1.2-fold in the presence of exogenous NAC (Figure 7A) whereas 80% GSH depletion was restored to 91% (Figure 7B) in the case of GO NPs exposure. As a result, NAC cotreatment caused increase in cell viability (Figure 7C) to 92% in case of IC_50_ of GO NPs and 81% in case of IC_50_ of ZnO NPs suggesting a better protection capacity of NAC from the toxicity of GO NPs.

## 4. Discussion

The hydrodynamic size of GO NPs were found to occur almost 4-times bigger in complete culture media (DMEM + 10% FBS) than were in TEM measurement suggesting significant agglomeration tendency. Particle agglomeration was significantly low in media than in water which is the indicative of media ingredients modifying NPs surfaces and colonization [48,49,50]. A variety of potentially interacting components in relevant aqueous fluids like culture media may influence the NPs native property. Serum proteins, for example, can greatly affect the surface property of NPs, influencing the dynamics of cellular interactions with NPs [48]. Cell viability in HUVECs were found to decrease in a concentration-dependent manner due to GO NPs when exposed for 48 h. Oxidative stress is considered as a major mechanism of toxicity behind that caused by NP exposure. Moreover, cells having bigger dimensions than are expected for cells like HUVECs that evidently present greater juxtapositions sites allowing direct nano-bio interactions may also result in toxicity that may be accounted as independently of, or in addition to, ROS [51,52]. In this study, cytotoxicity induced by GO NPs in HUVECs was highly correlated with membrane damage as demonstrated by LDH release, TBARS activity and BODIPY fluorescence. A higher green fluorescence of BODIPY reveals higher membrane damage caused by peroxidation reaction in cell membranes [30,31]. Interestingly, HUVECs compromised due to GO NPs remained adherent to the growth surface, whereas compromised cells due to ZnO NPs would detach from the growth surface, floating in the media, suggesting a different mode of mechanisms of cell death due to GO NPs and ZnO NPs (See cellular images in Figure 2).

Reactive oxidative species (ROS)—known to exert multiple and often contradictory effects in cells [53]—were found to be significantly elevated due to NPs of GO and ZnO. At excessive levels, ROS can initiate oxidative reactions in macromolecules, leading to cell cycle arrest, inflammation and finally, cell death that may be accompanied by apoptosis, necrosis, autophagy or by overlapping pathways [54,55,56]. ROS can cause significant exhaustion of cellular antioxidant resources like GSH that protects cells against ROS-induced damage. In this study, GO NPs tended to induce ROS more than that by ZnO NPs while ZnO NPs depleted GSH more than that by GO NPs. Induced ROS in a GSH-exhausted cell can provide a platform for the simultaneous occurrence of apoptosis and necrosis that may be intricately dependent on cell type, cellular energy store and extent of damage at the outer or inner interfaces of cells [57,58,59]. Seo et al. [60] reported the induction of ROS by GO NPs in abiotic aqueous solutions as well. Even ultra-low concentrations (2.5 µM/L) of GdCl_3_ salt have been shown to promote the production of ROS and cytokines in RAW 264.7 macrophage cells [61]. Mitochondrial-membrane potential (MMP) is the manifestation of induced ROS that is often considered a committed step in inducing death activating pathways [62]. Qualitative and quantitative data in the present report suggest that while both NPs caused significant loss in MMP, it was higher for GO NPs than for ZnO NPs.

NPs based on rare-earth elements—including those of gadolinium, cerium, neodymium—have been reported to induce autophagy in human macrophage THP-1 cells [63], in two human glioblastoma cells (*U*-87 MG and Mo59 K) [64] and in the cells of head and neck squamous cell carcinoma (HNSCC) [65]. Recently, gadolinium compound has also been reported to induce autophagy in the Mediterranean mussel Mytilus *galloprovincialis* [66]. In this study, acidic vesicles were detected significantly in high quantity in treated cells than in control HUVECs. Moreover, GO NPs caused significantly high autophagy than it was due to ZnO NPs at their respective IC_50s_. The functioning of nitric oxide production, angiogenesis and hemostasis/thrombosis brought out by endothelial cells including HUVECs are critically affected by autophagy and that any disruptions of autophagy can hamper the integrity of various tissue lining of these cells resulting in the pathogenesis of vascular diseases [67,68]. These results partly explain the mechanism of depositions of gadolinium in tissues undergoing MRI. Moreover, a recent study has reported significant rise in autophagy in the embryos of sea urchins due to the exposure of a gadolinium compound [66]. To date, data suggest that GO NPs exhibit comparatively robust tendency of inducing membrane damage, oxidative stress, mitochondrial dysfunction and autophagy in HUVECs while ZnO NPs exhibit these parameters in a more controlled manner at their respective IC_50s_. This different nature of mechanism becomes clearer when the mechanism of cell death is also under consideration. Some cells in GO NPs-treated group were identified that took up PI but lacked annexinV suggested a death mechanism independent of apoptosis in the case of GO NPs (see Figure 6). AnnexinV binding in other cells may be the result of late stage of disintegration and passive entry of annexinV inside cells [40], and that PI preferentially enters necrotic cells while is excluded from early apoptotic cells [41]. Another feature that suggest apoptosis-independent mechanism in the case of GO NPs is the size of nucleus with increased and dilated nuclear perimeter which is the characteristics of necrosis [42,43]. Recall that chromatin fragmentation in cells undergoing apoptosis is characterized by distinct stages of ring formation which is a continuous ring of condensed chromatin at the interior surface of the nuclear envelope followed by necklace condensation (discontinuities in ring) and nuclear collapse/disassembly (final apoptotic bodies) [69]. Caspase-3, an executioner enzyme in apoptosis, was significantly induced for GO NPs when compared to control group but was significantly low compared to ZnO NPs-induced caspase-3 activity.

Lee et al. reported two different modes of cells death in human endothelial cells (ECs) induced by NPs of silica [70] while another study also reported increased apoptosis and necrosis in HUVECs due to silica NPs [21]. Overall data in this study demonstrate that GO NPs activated both apoptosis and necrosis in HUVECs that may be the cumulative effect of higher MMP and autophagy induced by GO NPs. ZnO NPs that exclusively induced apoptosis may be due to higher intracellular GSH loss caused by it compared to GO NPs. It should be mentioned that silica NPs also induced autophagy in endothelial cells [71]. Interestingly, GO NPs were found to decrease MMP, but caused an increase in the expression of the Bax/Bcl_2_ ratio and apoptosis in human neuronal (SH-SY5Y) cells [72]. Large surface areas of cell membranes where peroxidation reactions may occur can lead to membrane damage favoring necrosis whereas apoptosis may also be simultaneously induced via induced ROS and GSH loss [73]. Other particles with fiber like appearances such as carbon nanotubes and asbestos have also been reported to induce membrane peroxidation, ROS and necroptosis in many cells including mesothelial [74,75]. Addition of antioxidant NAC, a GSH precursor [76], have significantly, but not completely, restored ROS and GSH imbalance caused by GO NPs suggesting oxidative stress a critical factor in the toxicity mechanism therein. Antioxidant NAC was also demonstrated to offer protection against the injury caused by gadolinium-based contrast agents (GBCAs) in experimental rats with chronic renal failure [77,78].

## 5. Conclusions

The present study demonstrated that the mechanism of damage due to GO NPs on HUVECs consists of membrane damage, oxidative stress, mitochondrial dysfunction and autophagy. ZnO NPs also induced these parameters, but in a more controlled manner. Moreover, while ZnO NPs clearly appeared to induce only apoptosis corroborating a robust GSH exhaustion, GO NPs revealed both apoptotic and necrotic potentials on HUVECs. The data partly explain the potential mechanism of toxicity due to gadolinium in vasculature and other tissues reported for gadolinium deposition following MRI.

## Figures and Tables

**Table 1:** Summary of physicochemical characterization data of GO nanoparticles.

Parameters	Physicochemical Properties
Color	White, powdery
Morphology by SEM and TEM	Nanofibers
Structural characterization by HR-TEM and XRD	Crystalline
Elemental composition by EDS	Gd and O, no other impurities detected
**Primary TEM Features**	
Diameter	9–23 nm (average particle diameter, 13.7 ± 6 nm)
Length	60–110 nm (average particle length, 54.8 ± 29 nm)
**DLS in Complete Culture Media**	
Hydrodynamic size	489 ± 43 nm
Zeta potential	−17 ± 4 eV
**DLS in Water**	
Hydrodynamic size	1104 ± 134 nm
Zeta potential	−9 ± 3 eV
